# Quantification of Effective Connectivity in the Brain Using a Measure of Directed Information

**DOI:** 10.1155/2012/635103

**Published:** 2012-05-16

**Authors:** Ying Liu, Selin Aviyente

**Affiliations:** Department of Electrical and Computer Engineering, Michigan State University, East Lansing, MI 48824, USA

## Abstract

Effective connectivity refers to the influence one neural system exerts on another and corresponds to the parameter of a model that tries to explain the observed dependencies. In this sense, effective connectivity corresponds to the intuitive notion of coupling or directed causal influence. Traditional measures to quantify the effective connectivity include model-based methods, such as dynamic causal modeling (DCM), Granger causality (GC), and information-theoretic methods. Directed information (DI) has been a recently proposed information-theoretic measure that captures the causality between two time series. Compared to traditional causality detection methods based on linear models, directed information is a model-free measure and can detect both linear and nonlinear causality relationships. However, the effectiveness of using DI for capturing the causality in different models and neurophysiological data has not been thoroughly illustrated to date. In addition, the advantage of DI compared to model-based measures, especially those used to implement Granger causality, has not been fully investigated. In this paper, we address these issues by evaluating the performance of directed information on both simulated data sets and electroencephalogram (EEG) data to illustrate its effectiveness for quantifying the effective connectivity in the brain.

## 1. Introduction

Neuroimaging technologies such as the electroencephalogram (EEG) make it possible to record brain activity with high temporal resolution and accuracy. However, current neuroimaging modalities display only local neural activity rather than large-scale interactions between different parts of the brain. Assessment of the large-scale interdependence between these recordings can provide a better understanding of the functioning of neural systems [1, 2]. Three kinds of brain connectivity are defined to describe such interactions between recordings: anatomical connectivity, functional connectivity, and effective connectivity [2]. Anatomical connectivity is the set of physical or structural connections linking neuronal units at a given time and can be obtained from measurements of the diffusion tensor [3, 4]. Functional connectivity captures the statistical dependence between scattered and often spatially remote neuronal units by measuring their correlations in either time or frequency domain. Effective connectivity describes how one neural system affects another [2, 4, 5], which can provide information about both the magnitude and the direction of the interaction.

The main approaches used to quantify the effective connectivity between two time series are model-based measures and information-theoretic measures [6]. Granger-causality-based methods and dynamic causal modeling [7] are two widely used model-based measures. Granger causality is a widely used measure to describe the causality between two time series. It defines a stochastic process **X** causing another process **Y** if the prediction of **Y** at the current time point, *Y*_*n*_, is improved when taking into account the past samples of **X**. This approach is appealing but gives rise to many questions on how to apply this definition to real data [8]. Granger causality has been mostly applied within a linear prediction framework using a multivariate autoregressive (MVAR) model yielding methods such as directed transfer function (DTF), partial directed coherence (PDC), and directed partial correlation [9–12]. For example, Hesse et al. applied time-varying Granger causality to EEG data and found that conflict situation generates directional interactions from posterior to anterior cortical sites [10]. Kamiński et al. applied DTF to EEG recordings of human brain during stage 2 sleep and located the main source of causal influence [11]. Schelter et al. employed PDC to EEG recordings from a patient suffering from essential tremor [13]. The extensions of Granger-causality-based methods, such as kernel Granger causality, generalized PDC (gPDC), and extended PDC (ePDC), have also found numerous applications in neuroscience [14–16]. However, Granger causality-based methods, especially those developed from MVAR models, are limited to capturing linear relations or require *a priori* knowledge about the underlying signal models [17]. These approaches may be misleading when applied to signals that are known to have nonlinear dependencies, such as EEG data [18]. DCM, on the other hand, can quantify nonlinear interactions by assuming a bilinear state space model. However, DCM requires *a priori* knowledge about the input to the system [7, 19] and is limited to a network with small size [4]. Thus, a model-free measure detecting both linear and nonlinear relationships is desired.

Information theoretic tools [20–22], such as transfer entropy [20], address the issue of model dependency and have found numerous applications in neuroscience [17, 23, 24]. “Transfer entropy” (TE) proposed by Schreiber computes causality as the deviation of the observed data from the generalized Markov condition and is defined as [20]

(1) $$\begin{array}{cc} & \text{TE}\left(X\to Y\right)\\ & \;\;=\underset{y_{n+1},y_{n-l+1:n},x_{n-m+1:n}}{\sum }p\left(y_{n+1}y_{n-l+1:n}x_{n-m+1:n}\right)\\ & \;\;\;\;\;\;\;\;\;\times \log\frac{p\left(y_{n+1}\;|\;y_{n-l+1:n}x_{n-m+1:n}\right)}{p\left(y_{n+1}\;|\;y_{n-l+1:n}\right)}, \end{array}$$

where *m* and *l* are the orders (memory) of the Markov processes **X** and **Y**, respectively. *p*(*y*_*n*+1_ *y*_*n*−*l*+1:*n*_ *x*_*n*−*m*+1:*n*_) is the joint probability of random variables (*Y*_*n*+1_, *Y*_*n*−*l*+1:*n*_, *X*_*n*−*m*+1:*n*_), where *Y*_*n*−*l*+1:*n*_ = (*Y*_*n*−*l*+1_,…, *Y*_*n*_) and *X*_*n*−*m*+1:*n*_ = (*X*_*n*−*m*+1_,…, *X*_*n*_). Sabesan et al. employed TE to identify the direction of information flow for the intracranial EEG data and suggested that transfer entropy plays an important role in epilepsy research [25]. Wibral et al. applied TE to magnetoencephalographic data to quantify the information flow in cortical and cerebellar networks [26]. Vicente et al. extended the definition of TE and measured the information flow from **X** to **Y** with a general time delay of *u*, that is, replaced *y*_*n*+1_ in the above equation with *y*_*n*+*u*_, and showed that TE has a better performance in detecting the effective connectivity for nonlinear interactions and signals affected by volume conduction such as real EEG/MEG recordings compared to linear methods [19]. The performance of transfer entropy depends on the estimation of transition probabilities, which requires the selection of order or memory of the Markov processes **X** and **Y** [25]. “Directed transinformation” (T) introduced by Saito and Harashima [21] measures the information flow from the current sample of one signal to the future samples of another signal given the past samples of both signals. Hinrichs et al. used this measure to analyze causal interactions in event-related EEG-MEG experiments [17]. However, this measure does not discriminate between totally dependent and independent processes [27]. Recently, directed information proposed by Marko [28] and later reformalized by Massey, Kramer, Tatikonda, and others have attracted attention for quantifying directional dependencies [22, 28–31]. Directed information theory has been mostly aimed towards the study of communication channels with feedback. In recent years, new theoretical developments motivated the use of this measure in quantifying causality between two time series. In particular, Amblard and Michel [31] recently showed how directed information and Granger causality are equivalent for linear Gaussian processes and proved key relationships between existing causality measures and the directed information. Therefore, there has been a growing interest in applying this measure to applications in signal processing, neuroscience, and bioinformatics. For example, it has been successfully used to infer genomic networks [32] and to quantify effective connectivity between neural spike data in neuroscience [31, 33, 34]. In order to detect both linear and nonlinear relationships, in this paper, we propose directed information as a powerful measure to quantify the effective connectivity in the brain.

The theoretical advantages of DI over existing measures have been noted in literature [31, 33, 34]. However, until now the benefits of using DI for capturing the effective connectivity in the brain through neurophysiological data have not been illustrated thoroughly and formally. In addition, because of the relationship between Granger causality and directed information, in this paper, we mainly focus on the comparison between these two measures and investigate the advantage of DI over Granger-causality-based model measures. Theoretical developments only proved the equivalence between these two measures for the case that the time series are distributed as Gaussian in a linear model. However, to date, there has not been much work that compares the actual performance of DI and Granger-causality-based measures for realistic signal models, including both linear and nonlinear interactions. Moreover, most applications of DI to real data have been limited to using either parametric density models for the data or making assumptions about the time dependencies such as assuming a first-order Markov chain and have not considered the difficulties associated with estimating DI from a finite sample size [35]. For complex systems, the computational complexity and the bias of the DI estimator increase with the length of the signal. The main contribution of this paper is to address these issues by evaluating the performance of DI and Granger-causality-based methods under a common framework without making any assumptions about the data distribution. In this paper, we first give a brief introduction to directed information and its computation based on nonparametric estimation methods. We propose a modified time-lagged directed information measure that simplifies the DI computation by reducing the order of the joint entropy terms while still quantifying the causal dependencies. We then evaluate the performance of DI for quantifying the effective connectivity for linear and nonlinear autoregressive models, linear mixing models, single source models, and dynamic chaotic oscillators in comparison to existing causality measures, in particular with Granger causality. Finally, we apply our method to EEG data to detect the effective connectivity in the brain.

## 2. Materials and Methods

### 2.1. Definitions and Notations

In this section, we will first review some common notations and information-theoretic definitions that will be used throughout this paper. Let **X** = *X* ^*n*^ = *X*_1:*n*_ = (*X*_1_,…, *X*_*n*_) be a random process with length *n* and *p*(*x*_1_,…, *x*_*n*_) = *p*(*x* ^*n*^) = *p*(*x*_1:*n*_) be the joint probability of random variables (*X*_1_,…, *X*_*n*_). *DX* ^*n*^ = *X* ^*n*−1^ = (0, *X*_1_,…, *X*_*n*−1_) will be used to define the time-delayed version of sequence *X* ^*n*^, which is also equivalent to *X*_1:*n*−1_.

Given two continuous random variables *X* and *Y*, the mutual information (MI) is defined as follows (All integrals in the paper are from −*∞* to +*∞* unless otherwise specified.):

(2) $$I\left(X;Y\right)=\int \int p\left(x,y\right)\log\frac{p\left(x,y\right)}{p_{x}\left(x\right)p_{y}\left(y\right)}dx\;dy,$$

where *p*(*x*, *y*) is the joint probability density function (pdf) of *X* and *Y*, and *p*_*x*_(*x*), *p*_*y*_(*y*) are the marginal pdfs of *X* and *Y*, respectively. *I*(*X*; *Y*) ≥ 0 with equality if and only if *X* and *Y* are independent [36]. In information theory, mutual information can be interpreted as the amount of uncertainty about *X* that can be reduced by observation of *Y*, or the amount of information *Y* can provide about *X*, that is, *I*(*X*; *Y*) = *H*(*X*) − *H*(*X* | *Y*). Since *I*(*X*; *Y*) ≥ 0, *H*(*X* | *Y*) ≤ *H*(*X*) with equality if and only if *X* and *Y* are independent; that is, conditioning reduces entropy [36].

For any three random variables *X*, *Y*, and *Z*, if the conditional distribution of *Z* depends only on *Y* and is conditionally independent of *X*, that is, *p*(*z* | *y*) = *p*(*z* | *yx*), then *X*, *Y*, and *Z* are said to form a Markov chain, denoted by *X* → *Y* → *Z*. In this case, the conditional mutual information between *X* and *Y* given *Z* defined as *I*(*X*; *Z* | *Y*) = *H*(*Z* | *Y*) − *H*(*Z* | *X*, *Y*) is equal to 0 [36].

### 2.2. Directed Information

Mutual information can be extended to random vectors or sequences *X* ^*N*^ and *Y* ^*N*^ as *I*(*X* ^*N*^; *Y* ^*N*^), where *I*(*X* ^*N*^; *Y* ^*N*^) = *H*(*X* ^*N*^) − *H*(*X* ^*N*^ | *Y* ^*N*^) = *H*(*Y* ^*N*^) − *H*(*Y* ^*N*^ | *X* ^*N*^). However, mutual information is a symmetric measure and does not reveal any directionality or causality between two random sequences. Massey addressed this issue by defining the directed information from a length *N* sequence *X* ^*N*^ = (*X*_1_,…, *X*_*N*_) to *Y* ^*N*^ = (*Y*_1_,…, *Y*_*N*_) [22] as follows:

(3) $$\begin{array}{cc} \text{DI}\left(X^{N}\to Y^{N}\right) & =H\left(Y^{N}\right)-H\left(Y^{N}||X^{N}\right)\\ & =\overset{N}{\underset{n=1}{\sum }}I\left(X^{n};Y_{n}\;|\;Y^{n-1}\right), \end{array}$$

where *H*(*Y* ^*N*^||*X* ^*N*^) is the entropy of the sequence *Y* ^*N*^ causally conditioned on the sequence *X* ^*N*^, and *H*(*Y* ^*N*^||*X* ^*N*^) is defined as

(4) $$H\left(Y^{N}||X^{N}\right)=\overset{N}{\underset{n=1}{\sum }}H\left(Y_{n}\;|\;Y^{n-1}X^{n}\right),$$

which differs from *H*(*Y* ^*N*^ | *X* ^*N*^) = ∑_*n*=1_^*N*^ *H*(*Y*_*n*_ | *Y* ^*n*−1^ *X* ^*N*^) in that *X* ^*n*^ replaces *X* ^*N*^ in each term on the right-hand side of (4), that is, only the causal influence of the time series **X** up to the current time sample *n* on the process **Y** is considered.

An alternative definition of the directed information is proposed by Tatikonda in terms of Kullback-Leibler (KL) divergence [30]. It shows that the difference between mutual information and directed information is the introduction of feedback in the definition of directed information [22, 30, 31]. Mutual information and directed information expressed by KL divergence are written as

(5) $$\begin{array}{c} I\left(X^{N};Y^{N}\right)=D_{\text{KL}}\left(p\left(x^{N},y^{N}\right)||p\left(x^{N}\right)p\left(y^{N}\right)\right),\\ \text{DI}\left(X^{N}\to Y^{N}\right)=D_{\text{KL}}\left(p\left(x^{N},y^{N}\right)||\overset{⟵}{p}\left(x^{N}\;|\;y^{N}\right)p\left(y^{N}\right)\right), \end{array}$$

where $\overset{⟵}{p}(x^{N}|y^{N})=\prod_{n=1}^{N}p(x_{n}|x^{n-1}y^{n-1})$ is the feedback factor influenced by the feedback in the system, that is, the probability that the input **X** at current time is influenced by the past values of both itself and **Y**. If there is no feedback, then *p*(*x*_*n*_ | *x* ^*n*−1^ *y* ^*n*−1^) = *p*(*x*_*n*_ | *x* ^*n*−1^) and $\overset{⟵}{p}(x^{N}|y^{N})=p(x^{N})$. In fact, $p(x^{N},y^{N})=\overset{⟵}{p}(x^{N}|y^{N})\vec{p}(y^{N}|x^{N})$, where $\vec{p}(y^{N}|x^{N})=\prod_{n=1}^{N}p(y_{n}|x^{n}y^{n-1})$ and is defined as the feedforward factor affected by the memory of the system. If the system is memoryless, then *p*(*y*_*n*_ | *x* ^*n*^ *y* ^*n*−1^) = *p*(*y*_*n*_ | *x*_*n*_).

### 2.3. Directed Information versus Granger Causality

Granger quantifies causality so that the time series *X* ^*N*^ causes *Y* ^*N*^ if the variance of the prediction error for **Y** at the present time is reduced by including past measurements from **X**. Based on Granger's definition of causality, Geweke introduced the Geweke's indices to quantify the causal linear dependencies under Gaussian assumptions [37]. Amblard and Michel proved that the directed information rate and Geweke's indices are equal for Gaussian processes [31] as indicated by

(6) $$\text{D}{\text{I}}_{\infty }\left(DX^{N}\to Y^{N}\right)=\frac{1}{2}\log\frac{\varepsilon_{\infty }^{2}\left(Y_{N}\;|\;Y^{N-1}\right)}{\varepsilon_{\infty }^{2}\left(Y_{N}\;|\;Y^{N-1}X^{N-1}\right)}=F_{X^{N}\to Y^{N}},$$

where *DX* ^*N*^ stands for the time-delayed sequence (0, *X*_1_,…, *X*_*N*−1_) with *N* being the length of the signal, DI_*∞*_(*X* ^*N*^ → *Y* ^*N*^) is the directed information rate; that is, DI_*∞*_(*X* ^*N*^ → *Y* ^*N*^) = lim⁡_*N*→*∞*_ *I*(*X* ^*N*^; *Y*_*N*_ | *Y* ^*N*−1^), *ε*_*∞*_^2^(*Y*_*N*_ | *Y* ^*N*−1^) = lim⁡_*N*→*∞*_ *ε* ^2^(*Y*_*N*_ | *Y* ^*N*−1^) is the asymptotic variance of the prediction residue when predicting *Y*_*N*_ from the observation of *Y* ^*N*−1^, and *F*_*X*^*N*^→*Y*^*N*^_ refers to the linear feedback measure from random processes *X* ^*N*^ to *Y* ^*N*^ defined by Geweke [37]. This equality shows that asymptotically the DI rate is equivalent to the gain in information by predicting **Y** using the past values of both **Y** and **X** compared to only using the past samples of **Y**, which is similar to the definition of Granger causality. Moreover, Amblard and Michel proved the equality of directed information and Granger's approach for multivariate time series in the case of Gaussian distributions [31]. 

### 2.4. Computation of Directed Information

The definition of DI for two length *N* sequences *X* ^*N*^ = (*X*_1_,…, *X*_*N*_) and *Y* ^*N*^ = (*Y*_1_,…, *Y*_*N*_) can also be rewritten in terms of the total change of joint entropy or mutual information along time as follows:

(7) $$\begin{array}{cc} \text{DI}\left(X^{N}\to Y^{N}\right) & =\overset{N}{\underset{n=1}{\sum }}I\left(X^{n};Y_{n}\;|\;Y^{n-1}\right)\\ & =\overset{N}{\underset{n=1}{\sum }}\left[H\left(X^{n}Y^{n-1}\right)-H\left(X^{n}Y^{n}\right)\right]+H\left(Y^{N}\right)\\ & =\overset{N}{\underset{n=1}{\sum }}\left[I\left(X^{n};Y^{n}\right)-I\left(X^{n};Y^{n-1}\right)\right]. \end{array}$$

From the above equations, we can observe that the computation of DI requires the estimation of joint probabilities of high-dimensional random variables over time. If *X*_*n*_ and *Y*_*n*_ are normally distributed, the joint entropy can be estimated based on the covariance matrices. However, for EEG data, the distribution is usually not Gaussian. The nonparametric entropy and mutual information estimators, such as plug-in estimator, m-spacing estimator, and Kozachenko and Leonenko (KL) estimator, have been extensively addressed in literature [38, 39]. In this paper, directed information estimation based on mutual information is used to estimate DI directly from EEG data by using adaptive partitioning method discussed in [39]. However, when the length of the signal increases, the computational complexity, the bias, and the variance of these estimators increase immensely with limited sample sizes. Methods that can reduce the dimension and simplify the computation of DI are needed.

In order to simplify the estimation of DI, we first clarify the connection between the definition of DI used in information theory and the definition as it applies to physical time series. In a physical recording system, if **X** starts to influence **Y** after *p*_1_ time points or with a delay of *p*_1_ samples, we need to record at least *N* + *p*_1_ time points to obtain *N* points of the time sequence **Y** that have been affected by **X**. The directed information rate from time series *X* ^*N*+*p*_1_^ to *Y* ^*N*+*p*_1_^ can be defined as [29]. We have

(8) $$\begin{array}{cc} & \text{D}{\text{I}}_{\infty }\left(X^{N+p_{1}}\to Y^{N+p_{1}}\right)\\ & \;\;=\underset{N+p_{1}\to \infty }{\lim}\frac{1}{N+p_{1}}\overset{N+p_{1}}{\underset{n=1}{\sum }}I\left(X^{n};Y_{n}\;|\;Y^{n-1}\right) \end{array}$$

(9) $$\begin{array}{cc} & \;\;=\underset{N+p_{1}\to \infty }{\lim}I\left(X^{N+p_{1}};Y_{N+p_{1}}\;|\;Y^{N+p_{1}-1}\right) \end{array}$$

(10) $$\begin{array}{cc} & \;\;=\underset{N+p_{1}\to \infty }{\lim}[H\left(Y_{N+p_{1}}\;|\;Y^{N+p_{1}-1}\right)\\ & \;\;\;\;\;\;\;-H\left(Y_{N+p_{1}}\;|\;X^{N+p_{1}}Y^{N+p_{1}-1}\right)] \end{array}$$

(11) $$\begin{array}{cc} & \;\;=\underset{N+p_{1}\to \infty }{\lim}[H\left(Y_{N+p_{1}}\;|\;Y_{p_{1}+1:N+p_{1}-1}\right)\\ & \;\;\;\;\;\;\;-H\left(Y_{N+p_{1}}\;|\;X^{N+p_{1}}Y_{p_{1}+1:N+p_{1}-1}\right)] \end{array}$$

(12) $$\begin{array}{cc} & \;\;=\underset{N+p_{1}\to \infty }{\lim}[H\left(Y_{N+p_{1}}\;|\;Y_{p_{1}+1:N+p_{1}-1}\right)\\ & \;\;\;\;\;\;\;-H\left(Y_{N+p_{1}}\;|\;X_{1:N}Y_{p_{1}+1:N+p_{1}-1}\right)] \end{array}$$

(13) $$\begin{array}{cc} & \;\;=\underset{N+p_{1}\to \infty }{\lim}I\left(X_{1:N};Y_{N+p_{1}}\;|\;Y_{p_{1}+1:N+p_{1}-1}\right) \end{array}$$

(14) $$\begin{array}{cc} & \;\;=\underset{N\to \infty }{\lim}\frac{1}{N}\overset{N}{\underset{n=1}{\sum }}I\left(X^{n};Y_{n+p_{1}}\;|\;Y_{p_{1}+1:n+p_{1}-1}\right) \end{array}$$

(15) $$\begin{array}{cc} & \;\;=\text{D}{\text{I}}_{\infty }\left(X_{1:N}\to Y_{p_{1}+1:p_{1}+N}\right), \end{array}$$

where (11) comes from the fact that *Y*_1:*p*_1__ is independent of *Y*_*N*+*p*_1__, and (12) is derived using the fact that *X*_*N*+1:*N*+*p*_1__ has no effect on *Y*_*N*+*p*_1__ because of the time delay *p*_1_ between these two time series. For two physical recordings **X** and **Y** with length *N* + *p*_1_ and a lag of *p*_1_, the last equation shows that DI rate for these two time series is equivalent to DI rate for two random processes with length *N* that are not synchronized in time. In fact, *Y*_*p*_1_+1:*p*_1_+*N*_ may be indexed as *Y*_1:*N*_ when using the information theoretic indexing, which indexes the signal not according to the physical time point but based on when the receiver receives its first piece of information. Therefore, directed information rate computed by using physical time indices is equivalent to the directed information rate using information theoretic indices for two systems that interact through a time delay. Moreover, when the length of the signal is long enough, the directed information value using both indices will be equivalent.

Once the definition of directed information is extended from random vectors to two physical time series, we propose a modified time-lagged DI to simplify the computation of DI, which is an extension of time-lagged DI proposed for every two samples of *X* ^*N*^ and *Y* ^*N*^ in [40] to general signal models. As we mentioned before, as the length *N* of the signal increases, the computational complexity, the bias, and the variance of estimating DI increase immensely with limited sample sizes. In addition, the directed information defined for the physical system is actually a DI with a lag of *p*_1_ samples over a time window with length *N*. Therefore, an intuitive way to simplify the computation is to apply DI with lag *p*_1_ over a small window. We first give the definition of time-lagged DI for two time series *X* ^*N*^ and *Y* ^*N*^ with length *N* at the *n*th time sample for a block of two time samples with a time delay of *p*_1_  (*n* > *p*_1_):

(16) $$\begin{array}{cc} & \text{D}{\text{I}}_{n}\left(X_{n-p_{1}}X_{n-p_{1}+1}\to Y_{n}Y_{n+1}\right)\\ & \;\;=I\left(X_{n-p_{1}};Y_{n}\right)+I\left(X_{n-p_{1}}X_{n-p_{1}+1};Y_{n+1}\;|\;Y_{n}\right)\\ & \;\;=H\left(X_{n-p_{1}}\right)+H\left(X_{n-p_{1}}X_{n-p_{1}+1}Y_{n}\right)+H\left(Y_{n}Y_{n+1}\right)\\ & \;\;\;-H\left(X_{n-p_{1}}Y_{n}\right)-H\left(X_{n-p_{1}}X_{n-p_{1}+1}Y_{n}Y_{n+1}\right), \end{array}$$

where *n* = *p*_1_ + 1,…, *N* − 1, *p*_1_ is the time lag between the two time series, and *N* is the length of the whole time series. Therefore, the total directed information over the whole time series in terms of the time-lagged DI can be simplified as [40] (the details of the derivation are given in [40]),

(17) $$\begin{array}{cc} & \text{DI}\left(X^{N}\to Y^{N}\right)\\ & \;\;=\overset{p_{1}}{\underset{n=1}{\sum }}I\left(X^{n};Y_{n}\;|\;Y^{n-1}\right)+\frac{N-p_{1}}{2\left(N-p_{1}-1\right)}\\ & \;\;\;\times \overset{N-1}{\underset{n=p_{1}+1}{\sum }}\text{D}{\text{I}}_{n}\left(X_{n-p_{1}}X_{n-p_{1}+1}\to Y_{n}Y_{n+1}\right). \end{array}$$

The time-lagged DI is equivalent to the original definition of DI when *p*_1_ is equal to the actual time delay of the system, the signals **X** and **Y** follow a single-order model, and *Y*_*n*_ only depends on one past sample of itself, *Y*_*n*−1_. However, these assumptions are not always true. Therefore, we propose the modified time-lagged DI to address these issues.

Consider a general Markov model, where *X* ^*N*^ and *Y* ^*N*^ are time series with a lag of *p*_1_ and *p*(*Y*_*n*_ | *X*_1:*n*−*p*_1__, *Y*_*p*_1_+1:*n*−1_) = *p*(*Y*_*n*_ | *X*_*n*−*p*_2_:*n*−*p*_1__, *Y*_*n*−*p*_3_:*n*−1_), where *p*_2_ ≥ *p*_1_, *p*_3_ ≥ 1, *p*_2_ is the order of the process **X**, and *p*_3_ is the order of the process **Y**. In this model, it is assumed that **X** starts to influence **Y** with a delay of *p*_1_ samples, and the order of the model is *p*_2_ − *p*_1_ + 1. When the length of the signal *N* is large enough, then (15) can be further simplified as

(18) $$\begin{array}{cc} \text{DI}\left(X^{N}\to Y^{N}\right) & =\text{DI}\left(X_{1:N-p_{1}}\to Y_{p_{1}+1:N}\right)\\ & =\overset{N}{\underset{n=p_{1}+1}{\sum }}I\left(X_{1:n-p_{1}},Y_{n}\;|\;Y_{p_{1}+1:n-1}\right)\\ & =\overset{N}{\underset{n=p_{1}+1}{\sum }}[H\left(Y_{n}\;|\;Y_{p_{1}+1:n-1}\right)\\ & \;\;\;\;-H\left(Y_{n}\;|\;X_{1:n-p_{1}}Y_{p_{1}+1:n-1}\right)]. \end{array}$$

Since *p*(*Y*_*n*_ | *X*_1:*n*−*p*_1__, *Y*_*p*_1_+1:*n*−1_) = *p*(*Y*_*n*_ | *X*_*n*−*p*_2_:*n*−*p*_1__, *Y*_*n*−*p*_3_:*n*−1_), *X*_1:*n*−*p*_2_−1_ *Y*_*p*_1_+1:*n*−*p*_3_−1_ → *X*_*n*−*p*_2_:*n*−*p*_1__, *Y*_*n*−*p*_3_:*n*−1_ → *Y*_*n*_ follows a Markov chain. According to Markov chain property,

(19) $$\begin{array}{cc} & I\left(X_{1:n-p_{2}-1}Y_{1:n-p_{3}-1};Y_{n}\;|\;X_{n-p_{2}:n-p_{1}}Y_{n-p_{3}:n-1}\right)\\ & \;\;=H\left(Y_{n}\;|\;X_{n-p_{2}:n-p_{1}}Y_{n-p_{3}:n-1}\right)\\ & \;\;\;-H\left(Y_{n}\;|\;X_{1:n-p_{1}}Y_{p_{1}+1:n-1}\right)\\ & \;\;=0, \end{array}$$

which means $\begin{array}{c} H(Y_{n}|X_{n-p_{2}:n-p_{1}}Y_{n-p_{3}:n-1})=H(Y_{n}|X_{1:n-p_{1}}Y_{p_{1}+1:n-1}) \end{array}$. Therefore,

(20) $$\begin{array}{cc} \text{DI}\left(X^{N}\to Y^{N}\right) & =\overset{N}{\underset{n=p_{1}+1}{\sum }}[H\left(Y_{n}\;|\;Y_{p_{1}+1:n-1}\right)\\ & \;\;\;\;-H\left(Y_{n}\;|\;X_{1:n-p_{1}}Y_{p_{1}+1:n-1}\right)]\\ & =\overset{N}{\underset{n=p_{1}+1}{\sum }}[H\left(Y_{n}\;|\;Y_{p_{1}+1:n-1}\right)\\ & \;\;\;\;-H\left(Y_{n}\;|\;X_{n-p_{2}:n-p_{1}}Y_{n-p_{3}:n-1}\right)]\\ & \leq \overset{N}{\underset{n=p_{1}+1}{\sum }}[H\left(Y_{n}\;|\;Y_{n-p_{3}:n-1}\right)\\ & \;\;\;\;-H\left(Y_{n}\;|\;X_{n-p_{2}:n-p_{1}}Y_{n-p_{3}:n-1}\right)]\\ & =\overset{N}{\underset{n=p_{1}+1}{\sum }}I\left(X_{n-p_{2}:n-p_{1}};Y_{n}\;|\;Y_{n-p_{3}:n-1}\right), \end{array}$$

where the second equality is using the Markov property, and the inequality comes from the fact that conditioning reduces entropy. For a general Markov model, where *X* ^*N*^ and *Y* ^*N*^ are stationary statistical processes without instantaneous interaction, such as *p*(*Y*_*n*_ | *X*_1:*n*−*p*_1__, *Y*_*p*_1_+1:*n*−1_) = *p*(*Y*_*n*_ | *X*_*n*−*p*_2_:*n*−*p*_1__, *Y*_*n*−*p*_3_:*n*−1_), the modified time-lagged directed information (MDI) is defined as the upper bound of DI:

(21) $$\begin{array}{cc} & \text{MDI}\left(X^{N}\to Y^{N}\right)\\ & \;\;=\overset{N}{\underset{n=p+1}{\sum }}I\left(X_{n-p}\cdots X_{n-1};Y_{n}\;|\;Y_{n-p}\cdots Y_{n-1}\right), \end{array}$$

where we let *p*_1_ = 1, *p* = max⁡(*p*_2_, *p*_3_) to reduce the number of parameters. Note that letting *p*_1_ = 1 does not lose any of the information flow compared to using the actual time delay, *p*_1_ > 1. The only drawback of letting *p*_1_ = 1 is that the computational complexity of estimating the joint entropies increases since the length of the window to compute MDI increases and the dimensionality increases. The main reason why we let *p*_1_ = 1 is because estimating the actual value for the delay accurately is not practical when the amount of data is limited. In a lot of similar work such as in [19], different values of *p*_1_ are tested to choose the best one which is not computationally efficient either.

According to (20), modified time-lagged directed information is the upper bound of directed information, that is, MDI ≥ DI. Moreover, MDI is a more general extension of time-lagged DI introduced in our previous work and has two major advantages. First, MDI considers the influence of multiple past samples of **Y** on the DI value. Second, it takes into account models with multiple orders; that is, **Y** is influenced by different time lags of **X**. The modified time-lagged directed information extends the length of the window from 2 to *p*, which is closer to the actual information flow. When **X** and **Y** are normally distributed, the computational complexity of the MDI is *O*(*p* ^3^ *N*) and the computational complexity of the original definition of DI is *O*(*N* ^4^) (using LU decomposition [41]). Therefore, the computation of MDI is more efficient than that of the original definition of DI.

### 2.5. Order Selection

For the implementation of MDI, we need to determine the maximum order of the model *p*. Criterions such as Akaike's final prediction error (FPE) can be used to determine the order of the signal model *p*. However, this criterion is based on the assumption that the original signal follows a linear AR model and may lead to false estimation of the order when the underlying signal model is nonlinear. Therefore, model-free order selection methods, such as the embedding theorem [42], are needed. For the simplification of computation or parameter estimation, we are only interested in a limited number of variables that can be used to describe the whole system. Suppose we have a time series (*X*_1_,…, *X*_*n*_), and the time-delay vectors can be reconstructed as (*X*_*n*_, *X*_*n*−*τ*_, *X*_*n*−2*τ*_,…, *X*_*n*−(*d*−1)*τ*_). Projecting the original system to this lower-dimensional state space-depends on the choice of *d* and *τ*, and the optimal embedding dimension *d* is related to the order of the model *p* = *d* [19]. A variety of measures such as mutual information can be used to determine *τ*. For discrete time signals, usually the best choice of *τ* is 1 [43]. To determine *d*, Cao criterion based on the false nearest neighbor procedure [19] is used to determine the local dimension. The underlying concept of nearest neighbor is that if *d* is the embedding dimension of a system, then any two points that stay close in the *d*-dimensional reconstructed space are still close in the (*d* + 1)-dimensional reconstructed space; otherwise, these two points are false nearest neighbors [19, 43]. The choice of *d*, that is, the model order *p*, is important for DI estimation. If *d* is too small, we will lose some of the information flow from **X** to **Y**. If it is too large, the computational complexity of MDI will be very high, causing the bias and the variance of the estimators to increase.

### 2.6. Normalization and Significance Test

Since DI(*X* ^*N*^ → *Y* ^*N*^) + DI(*Y* ^*N*^ → *X* ^*N*^) = *I*(*X* ^*N*^; *Y* ^*N*^) + DI(*X* ^*N*^ → *Y* ^*N*^||*DX* ^*N*^) and DI(*X* ^*N*^ → *Y* ^*N*^) = DI(*DX* ^*N*^ → *Y* ^*N*^) + DI(*X* ^*N*^ → *Y* ^*N*^||*DX* ^*N*^) [29], then

(22) $$\begin{array}{cc} & \text{DI}\left(X^{N}\to Y^{N}\right)+\text{DI}\left(Y^{N}\to X^{N}\right)\\ & \;\;=\text{DI}\left(DX^{N}\to Y^{N}\right)+\text{DI}\left(X^{N}\to Y^{N}||DX^{N}\right)\\ & \;\;\;+\text{DI}\left(DY^{N}\to X^{N}\right)+\text{DI}\left(Y^{N}\to X^{N}||DY^{N}\right). \end{array}$$

Therefore,

(23) $$\begin{array}{cc} & \text{DI}\left(DX^{N}\to Y^{N}\right)+\text{DI}\left(DY^{N}\to X^{N}\right)\\ & \;+\text{DI}\left(Y^{N}\to X^{N}||DY^{N}\right)=I\left(X^{N};Y^{N}\right), \end{array}$$

where DI(*Y* ^*N*^ → *X* ^*N*^||*DY* ^*N*^) = DI(*X* ^*N*^ → *Y* ^*N*^||*DX* ^*N*^) indicating the instantaneous information exchange between processes **X** and **Y**. For a physical system without instantaneous causality, that is, *I*(*X* ^*N*^ → *Y* ^*N*^||*DX* ^*N*^) = 0, then DI(*X* ^*N*^ → *Y* ^*N*^) + DI(*Y* ^*N*^ → *X* ^*N*^) = *I*(*X* ^*N*^; *Y* ^*N*^) and 0 ≤ DI(*X* ^*N*^ → *Y* ^*N*^) ≤ *I*(*X* ^*N*^; *Y* ^*N*^) < *∞*. A normalized version of DI, which maps DI to the [0,1] range, is used for comparing different interactions,

(24) $$\begin{array}{cc} \rho_{\text{DI}}\left(X^{N}\to Y^{N}\right) & =\frac{\text{DI}\left(X^{N}\to Y^{N}\right)}{I\left(X^{N};Y^{N}\right)}\\ & =\frac{\text{DI}\left(X^{N}\to Y^{N}\right)}{\text{DI}\left(X^{N}\to Y^{N}\right)+\text{DI}\left(Y^{N}\to X^{N}\right)}, \end{array}$$

where for a unidirectional system **X** → **Y** with no instantaneous interaction between **X** and **Y**, *ρ*_DI_(*X* ^*N*^ → *Y* ^*N*^) = 1 and *ρ*_DI_(*Y* ^*N*^ → *X* ^*N*^) = 0; otherwise, if there is no causal relationship between the two signals, the values of *ρ*_DI_(*X* ^*N*^ → *Y* ^*N*^) and *ρ*_DI_(*Y* ^*N*^ → *X* ^*N*^) are very close to each other.

In order to test the null hypothesis of noncausality, the causal structure between **X** and **Y** is destroyed. For each process with multiple trials, we shuffle the order of the trials of the time series **X** 100 times to generate new observations **X**_m_*, *m* = 1,…, 100. In this way, the causality between **X** and **Y** for each trial is destroyed, and the estimated joint probability changes [44]. We compute the DI for each pair of data (**X**_m_* and **Y**). A threshold is obtained at a *α* = 0.05 significance level such that 95% of the directed information for randomized pairs of data (DI(**X**_m_* → **Y**)) is less than this threshold. If the DI value of the original pairs of data is larger than this threshold, then it indicates there is significant information flow from **X** to **Y**.

### 2.7. Simulated Data

To test the validity and to evaluate the performance of DI for quantifying the effective connectivity, we generate five different simulations. We use these simulation models to compare DI with classical Granger causality (GC) for quantifying causality of both linear and nonlinear autoregressive models, linear mixing models, single source models, and Lorenz systems. The Matlab toolbox developed by Seth is used to compute the GC value in the time domain. GC is also normalized to the [0,1] range for comparison purposes [45]. The performance of GC depends on the length of the signal, whereas the performance of DI relies on the number of realizations of time series. Therefore, for each simulation, the length of the generated signal for implementing GC is equal to the number of realizations for DI. The significance of DI values are evaluated by shuffling along the trials, while the significance of GC values are evaluated by shuffling along the time series.

**Example 1 (Multiple Order Bivariate Linear Autoregressive Model)**

In this example, we evaluate the performance of DI on a general bivariate linear model,

(25) $$\begin{array}{c} X\left(n\right)=\overset{p_{4}}{\underset{i=1}{\sum }}\alpha_{i}X\left(n-i\right)+\sigma_{x}\eta_{x}\left(n-1\right), \end{array}$$

(26) $$\begin{array}{c} Y\left(n\right)=\overset{p_{3}}{\underset{i=1}{\sum }}\beta_{i}Y\left(n-i\right)+\gamma \overset{p_{2}}{\underset{i=p_{1}}{\sum }}X\left(n-i\right)+\sigma_{y}\eta_{y}\left(n-1\right). \end{array}$$

In this bivariate AR model with a delay *p*_1_ and order *p*_2_ − *p*_1_ + 1, *γ* controls the coupling strength between the signals **X** and **Y**. The initial values of **X** and **Y** and the noise *η*_*x*_ and *η*_*y*_ are all generated from a Gaussian distribution with mean 0 and standard deviation 1. All coefficients (*α*_*i*_, *β*_*i*_, *σ*_*x*_, and *σ*_*y*_) are generated from Gaussian distributions with zero mean and unit variance with unstable systems being discarded. To evaluate the performance of directed information, we generate the bivariate model 4096 times with the same parameters but different initial values. *γ* is varied from 0.1 to 1 with a step size of 0.1, *p*_1_ = 1 and *p*_2_ = *p*_3_ = *p*_4_ = 5; that is, **Y** is influenced by **X** through multiple time lags. Without loss of generality, we repeat the simulation 10 times, and average DI(*X* ^*N*^ → *Y* ^*N*^) and DI(*Y* ^*N*^ → *X* ^*N*^) over 10 simulations for different *γ* values. For each simulation, the threshold is evaluated by trial shuffling, and the average threshold is obtained. For GC, the length of the generated signal is chosen as 4096, which is the same as the number of realizations for DI. The GC values in two directions and the corresponding thresholds at the 5% significance level are obtained.

**Example 2 (Multiple-Order Bivariate Nonlinear Autoregressive Model)**

In this example, we evaluate the performance of DI on a general bivariate nonlinear model

(27) $$\begin{array}{cc} X\left(n\right) & =\overset{p_{4}}{\underset{i=1}{\sum }}\alpha_{i}X\left(n-i\right)+\sigma_{x}\eta_{x}\left(n-1\right), \end{array}$$

(28) $$\begin{array}{cc} Y\left(n\right) & =\overset{p_{3}}{\underset{i=1}{\sum }}\beta_{i}Y\left(n-i\right)\\ & \;+\gamma \overset{p_{2}}{\underset{i=p_{1}}{\sum }}\frac{1}{1+\exp\left(b_{1}+b_{2}X\left(n-i\right)\right)}\\ & \;+\sigma_{y}\eta_{y}\left(n-1\right). \end{array}$$

For this bivariate nonlinear AR model, the setting for the coupling strength *γ* and the generation of **X**, **Y**, *η*_*x*_, *η*_*y*_, *α*_*i*_, *β*_*i*_, *σ*_*x*_, *σ*_*y*_, *p*_1_, *p*_2_, *p*_3_, and *p*_4_ are the same as in Example 1. **Y** and **X** interact nonlinearly through the sigmoid function. Parameters of this function *b*_1_ and *b*_2_ control the threshold level and slope of the sigmoidal curve, respectively. We set *b*_1_ = 0 and *b*_2_ = 50. DI value and its threshold are averaged over 10 simulations for different *γ*. The GC values in two directions and the corresponding thresholds at 5% significance level are obtained.

**Example 3 (Linear Mixing Model)**

In this example, we test the effectiveness of DI in inferring effective connectivity when there is linear mixing between these two signals. Linear instantaneous mixing is known to exist in human noninvasive electrophysiological measurements such as EEG or MEG. Instantaneous mixing from coupled signals onto sensor signals by the measurement process degrades signal asymmetry [19]. Therefore, it is hard to detect the causality between the two signals. For unidirectional coupled signal pairs **X** → **Y** described in (25) to (28), we create two linear mixtures **X**_*ϵ*_ and **Y**_*ϵ*_ as follows:

(29) $$\begin{array}{c} X_{\epsilon }\left(n\right)=\left(1-\epsilon \right)X\left(n\right)+\epsilon Y\left(n\right),\\ Y_{\epsilon }\left(n\right)=\epsilon X\left(n\right)+\left(1-\epsilon \right)Y\left(n\right), \end{array}$$

where *ϵ* controls the amount of linear mixing and is varied from 0.05 to 0.45 with a step size of 0.05, and *γ* is fixed to 0.8 for both models. When *ϵ* = 0.5, the two signals are identical. Both DI and GC are used to quantify the information flow between **X**_*ϵ*_ and **Y**_*ϵ*_ in the two directions.

**Example 4 (Single-Source Model)**

A single source is usually observed on different signals (channels) with individual channel noises [19], which is common in EEG signals due to the effects of volume conduction. In this case, false positive detection of effective connectivity occurs for methods such as Granger causality [46], which means that GC has low specificity. We generate two signals **X**_*ϵ*_ and **Y**_*ϵ*_ as follows to test the specificity of DI when there is no significant information flow from one signal to the other signal. We have

(30) $$\begin{array}{c} S\left(n\right)=\overset{p_{4}}{\underset{i=1}{\sum }}\alpha_{i}S\left(n-i\right)+\eta_{S}\left(n\right),\\ X_{\epsilon }\left(n\right)=S\left(n\right),\\ Y_{\epsilon }\left(n\right)=\left(1-\epsilon \right)S\left(n\right)+\epsilon \eta_{Y}\left(n\right), \end{array}$$

where *S*(*n*) is the common source generated by an autoregressive model, order *p*_4_ = 5, *α*_*i*_ and *η*_*S*_(*n*) are generated from a Gaussian distribution with mean 0 and standard deviation 1. *S*(*n*) is measured on both sensors **X**_*ϵ*_ and **Y**_*ϵ*_. **Y**_*ϵ*_ is further corrupted by independent Gaussian noise *η*_*Y*_(*n*) with 0 mean and unit variance. *ϵ* controls the signal to noise ratio (SNR) in **Y**_*ϵ*_ and is varied from 0.1 to 0.9 with a step size of 0.1, corresponding to SNR in the range of −19 ~ 19 dB.

**Example 5 (Nonlinear Dynamic System)**

In this example, we illustrate the applicability of DI to coupled Lorenz oscillators with a certain delay. The Lorenz oscillator is a three-dimensional dynamic system that exhibits chaotic behavior. Synchronization of two Lorenz systems has been widely investigated for the analysis of EEG data because the dynamic interactions related to the behavior of the cortex can be exemplified by these coupled systems [47]. In the following, we examined two asymmetric coupled Lorenz oscillators (*X*_1_, *Y*_1_, *Z*_1_) and (*X*_2_, *Y*_2_, *Z*_2_) as follows [48]:

(31) $$\begin{array}{c} {\dot{X}}_{1}\left(t\right)=-A\left(X_{1}\left(t\right)-Y_{1}\left(t\right)\right),\\ {\dot{Y}}_{1}\left(t\right)=RX_{1}\left(t\right)-Y_{1}\left(t\right)-X_{1}\left(t\right)Z_{1}\left(t\right),\\ {\dot{Z}}_{1}\left(t\right)=X_{1}\left(t\right)Y_{1}\left(t\right)-BZ_{1}\left(t\right),\\ {\dot{X}}_{2}\left(t\right)=-A\left(X_{2}\left(t\right)-Y_{2}\left(t\right)\right)+\beta X_{1}\left(t-t_{p}\right),\\ {\dot{Y}}_{2}\left(t\right)=RX_{2}\left(t\right)-Y_{2}\left(t\right)-X_{2}\left(t\right)Z_{2}\left(t\right),\\ {\dot{Z}}_{2}\left(t\right)=X_{2}\left(t\right)Y_{2}\left(t\right)-BZ_{2}\left(t\right), \end{array}$$

where each equation is a first-order differential equation. *A* = 10, *R* = 28, *B* = 8/3, and *t*_*p*_ = 0.02 represents the time delay between two coupled components of these two oscillators, that is, **X**_1_ and **X**_2_. *β* corresponds to the coupling strength and is varied from 0.1 to 1 with a step size of 0.2. The differential equations are numerically integrated with a time step of 0.01 using Euler's method [49], corresponding to a delay of 2 time samples between *X*_1_ and *X*_2_. The initial conditions of these six components are randomly generated from a Gaussian distribution with zero mean and unit variance. We generate 100 samples, and the first 90 samples are discarded to eliminate the initial transients. We compute the information flow in two directions over 10 time points, and the significance of the obtained DI value is verified by trial shuffling.

### 2.8. Biological Data

In this paper, we examine EEG data from ten undergraduates at Michigan State University drawn from an ongoing study of relationships between the error-related negativity (ERN) and individual differences (Participants for the present analysis were drawn from samples reported on in [50, 51]) such as worry and anxiety. ERN is a brain potential response that occurs following performance errors in a speeded reaction time task [52]. All participants retained for analysis make at least six errors for computation of stable ERNs, as in [53]. Participants complete a letter version of the Eriksen Flanker task [52]. Stimuli are presented on a Pentium R Dual Core computer, using Presentation software (Neurobehavioral systems, Inc.) to control the presentation and timing of stimuli, the determination of response accuracy, and the measurement of reaction times. Continuous electroencephalographic activity is recorded by 64 Ag-AgCl electrodes placed in accordance with the 10/20 system. Electrodes are fitted in a BioSemi (BioSemi, Amsterdam, The Netherlands) stretch-lycra cap. All bioelectric signals are digitized at 512 Hz using ActiView software (BioSemi). For each subject, EEG data are preprocessed by the spherical spline current source density (CSD) waveforms to sharpen event-related potential (ERP) scalp topographies and eliminate volume conduction [54]. In addition, a bandpass filter is used to obtain signals in the theta band. In this study we focus on 33 electrodes corresponding to the frontal, central, and parietal regions of the brain. For each pair of 33 electrodes **X** and **Y** for each subject, the effective connectivity is quantified by computing the modified time-lagged DI over 70 trials and a model order of *p* in the theta band. The model order or the length of the time window *p* is determined by the Cao Criterion. We also apply Granger causality to the same data and compare its performance with directed information.

Previous work indicates that there is increased synchronization associated with ERN for the theta frequency band (4–8 Hz) and ERN time window 25–75 ms after the response for error responses (ERN) in the anterior cingulate cortex (ACC), in particular between the lateral prefrontal cortex (lPFC) and medial prefrontal cortex (mPFC) [55]. In this paper, we wish to verify these existing findings using the proposed DI measure and to further infer the directional causality underlying these dependencies.

## 3. Results and Discussion

In this section, we first evaluate the effectiveness of directed information on quantifying both linear and nonlinear causal relationships through simulated data and compare the performance of directed information with GC. We then apply the directed information to real EEG data to reveal the pair-wise information flow in the brain. 

### 3.1. Simulated Data

**Example 1 (Multiple-Order Bivariate Linear Autoregressive Model)**

**Figure 1:**
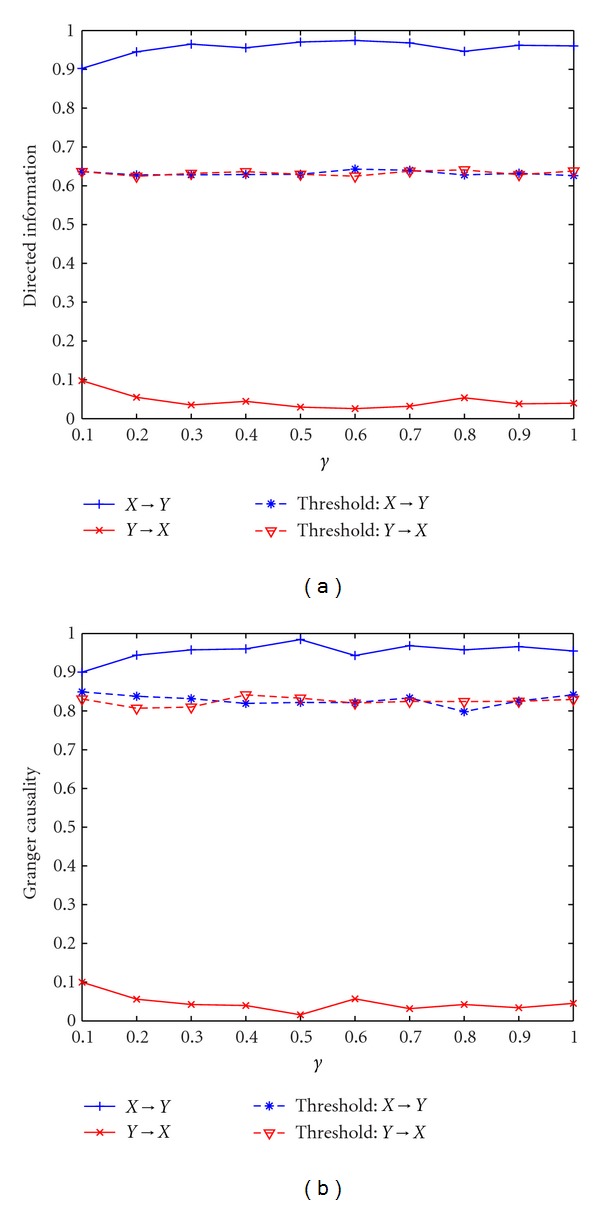
Application of directed information and Granger causality to bivariate linear autoregressive model. (a) Directed information with different *γ*. (b) Granger causality with different *γ*.

In this example, the DI value in two directions averaged across 10 simulations with different *γ* is shown in Figure 1(a). The performance of GC is shown in Figure 1(b). The estimated order of the model is *p* = 5, which is in accordance with the simulation model. *γ* controls the coupling strength between **X** and **Y**. We observe that DI(*X* ^*N*^ → *Y* ^*N*^) is significant for all values of *γ*. On the contrary, DI(*Y* ^*N*^ → *X* ^*N*^) is less than the threshold, which indicates the acceptance of the null hypothesis that there is no significant causal information flow from **Y** to **X**. Since GC uses a linear autoregressive framework for quantifying causality; in this example, GC detects the causality relationship between **X** and **Y** successfully; that is, the information flow from **X** to **Y** is significant for all *γ* while it is insignificant for the opposite direction. It is also interesting to note that GC and DI exhibit similar behavior across different values of *γ*, indicating the equivalency of the two measures for linear Gaussian signal models.

**Example 2 (Multiple-Order Bivariate Nonlinear Autoregressive Model)**

**Figure 2:**
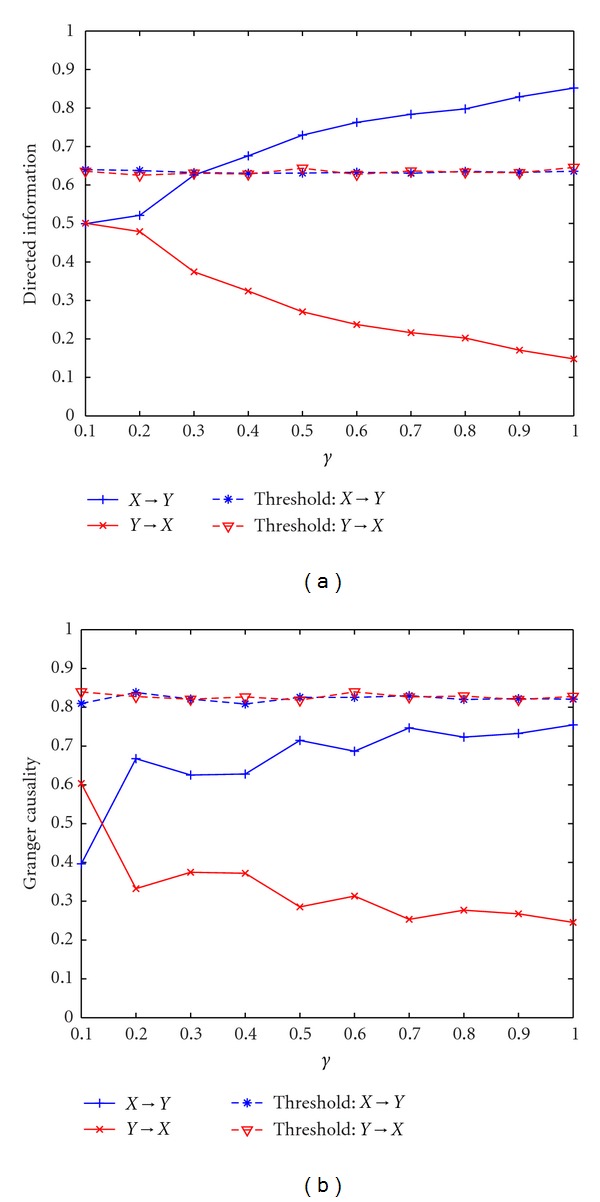
Application of directed information and Granger causality to bivariate nonlinear autoregressive model. (a) Directed information with different *γ*. (b) Granger causality with different *γ*.

In this example, the performance of DI and GC for the nonlinear autoregressive model in (27) and (28) averaged across 10 simulations with different *γ* are evaluated as shown in Figure 2. The estimated order of the model is 5. We observe that when *γ* is less than 0.3, the coupling strength between **X** and **Y** is weak and the DI value in both directions is not significant. As *γ* increases, DI(*X* ^*N*^ → *Y* ^*N*^) increases and becomes significant. DI(*Y* ^*N*^ → *X* ^*N*^) decreases with increasing *γ* and is still less than the threshold as expected. The results indicate increased unidirectional information flow from **X** to **Y** with increasing *γ* and show that detecting the information flow in nonlinear processes is more difficult especially when the coupling strength is low. GC fails to detect the information flow from **X** to **Y** for all *γ*. Since GC is implemented in a linear framework, the estimated order and the model itself do not match with the nonlinearity of the signal. Therefore, it cannot detect nonlinear causality.

**Example 3 (Linear Mixing Model)**

**Figure 3:**
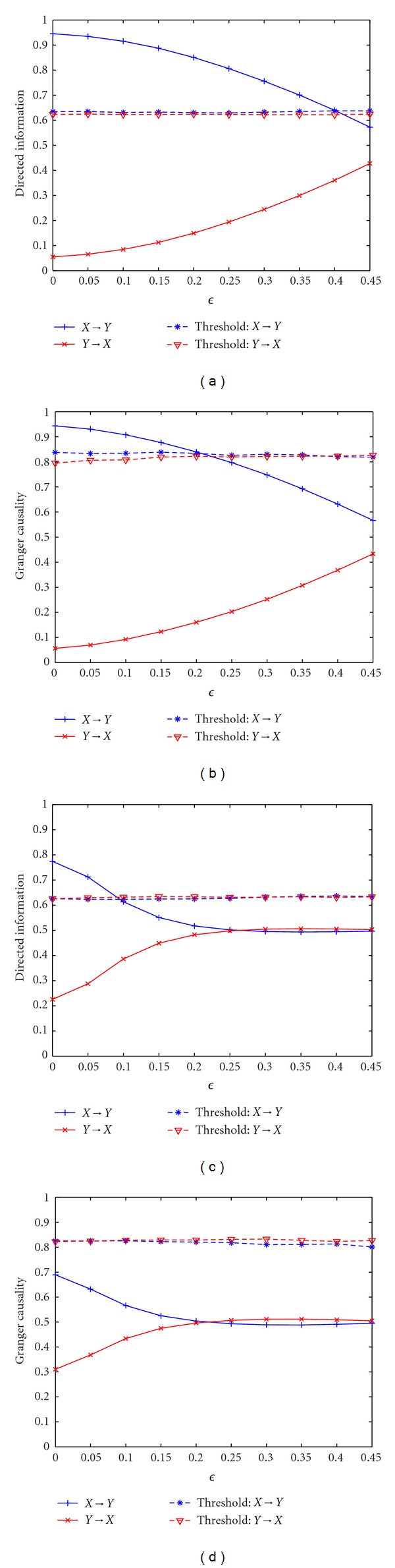
Application of directed information and Granger causality to linear mixing for both linear and nonlinear autoregressive models. (a) Directed information with different *ϵ* for the linear mixing of linear AR model. (b) Granger causality with different *ϵ* for the linear mixing of linear AR model. (c) Directed information with different *ϵ* for the linear mixing of nonlinear AR model. (d) Granger causality with different *ϵ* for the linear mixing of nonlinear AR model.

For this example, the DI value and GC value averaged across 10 simulations with changing linear mixing coefficient *ϵ* for both linear and nonlinear AR models are shown in Figure 3. The estimated order of the model is 5 as before. When *ϵ* = 0.5, the two observed mixing signals are identical, and we expect to see no significant information flow in the two directions. We observe that, for the linear AR model, directed information detects the causality between **X**_*ϵ*_ and **Y**_*ϵ*_ when *ϵ* is smaller than 0.4. When *ϵ* is larger than 0.4, the causality between **X**_*ϵ*_ and **Y**_*ϵ*_ is hard to detect because of the strong mixing; that is, **X**_*ϵ*_ and **Y**_*ϵ*_ are almost identical, and the information flow in both directions becomes insignificant. Compared to DI, GC only detects the causality from **X**_*ϵ*_ to **Y**_*ϵ*_ when the mixing is weak (*ϵ* < 0.2), indicating that GC is more vulnerable to linear mixing. It is probably due to the fact that GC is sensitive to the mixture of signals, and the assumed signal model does not match with the original signal [46]. For the nonlinear AR model, DI fails to detect causality when *ϵ* is larger than 0.1, which indicates that linear mixing of nonlinear source models makes it harder to detect effective connectivity compared to mixing of linear source models. On the other hand, GC fails to detect any causality even when *ϵ* = 0 since it cannot detect nonlinear interactions.

**Example 4 (Single-Source Model)**

**Figure 4:**
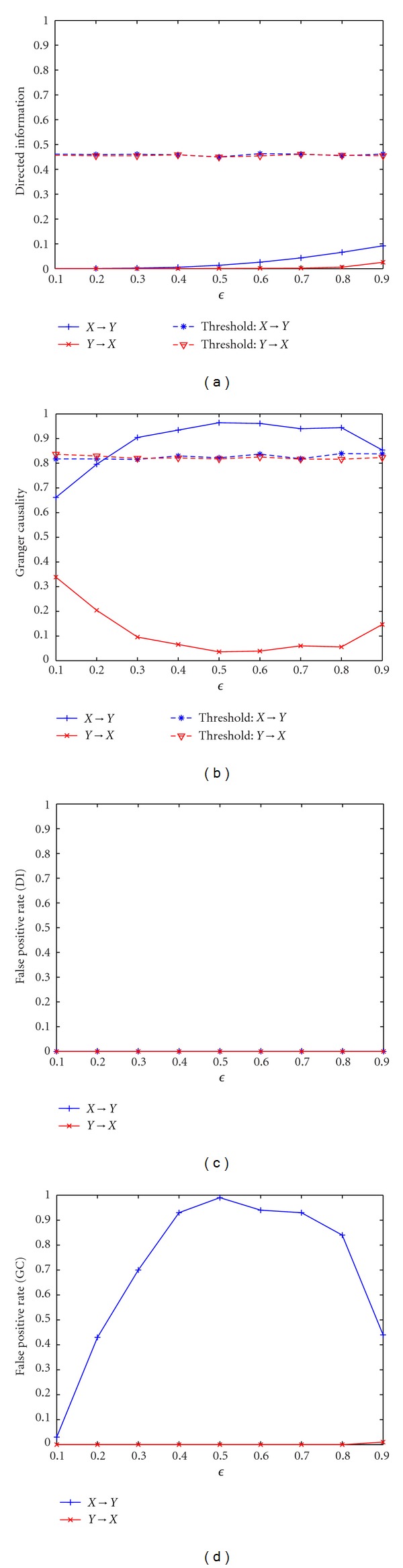
Application of directed information and Granger causality to single source model. (a) Directed information with different *ϵ* for the single source model. (b) Granger causality with different *ϵ* for the single source model. (c) False positive rate for directed information with different *ϵ* for the single source model. (d) False positive rate for Granger causality with different *ϵ* for the single source model.

We use the single source model to test the specificity of DI. The DI value and GC value averaged across 100 simulations for changing *ϵ* for a single source model are shown in Figure 4. The estimated order of the model is 5 as before. In addition, the false positive rate using both DI and Granger causality with increasing *ϵ* is also calculated. We observe that the information flow in two directions using DI is less than the threshold for all values of *ϵ*, which indicates the acceptance of the null hypothesis that there is no significant causal information flow from *X* to *Y* or *Y* to *X*. Note that DI is normalized by the mutual information. For a common source model, the instantaneous information exchange between *X* and *Y* contributes mostly to the mutual information between **X** and **Y**. Thus, according to (23), DI(*DX* ^*N*^ → *Y* ^*N*^) and DI(*DY* ^*N*^ → *X*) normalized by mutual information are close to 0 and less than the threshold from the randomized data pairs. The false positive rate of DI is 0 for all *ϵ*. Therefore, DI is able to discriminate between instantaneous mixing from actual causality and is very robust to noise. For GC, when *ϵ* is small (<0.2) or large (>0.9), the value of GC is less than or very close to the threshold in both directions thus indicating that there is no causal information flow between the two processes. However, GC fails to accept the null hypothesis when *ϵ* is between 0.3 to 0.9 and detects a nonexisting effective connectivity. GC reaches its maximum value when *ϵ* = 0.5. This is due to the fact that GC is close to 0 when two processes *X* and *Y* are independent or identical, that is, when *ϵ* = 1 and *ϵ* = 0. Based on the definition of GC, the prediction of **Y** at the current time point will not be improved by taking into account the past samples of **X** for these processes [26]. Therefore, as *ϵ* increases from 0 to 0.5, **X** becomes the most different from **Y**; therefore, it can provide more new information about **Y** and the GC increases. As *ϵ* increases from 0.5 to 1, **X** becomes independent of **Y**, and the GC decreases. The false positive rate of GC is not equal to 0 for all values of *ϵ*, which indicates that it has lower specificity compared to DI. Therefore, GC is not robust to the effect of a common source and may infer false positive effective connectivity. This simulation indicates that DI is more sensitive and discriminative about the information flow patterns in the presence of volume conduction, which means it is a more promising method to capture the effective connectivity for real EEG data.

**Example 5 (Nonlinear Dynamic System)**

**Figure 5:**
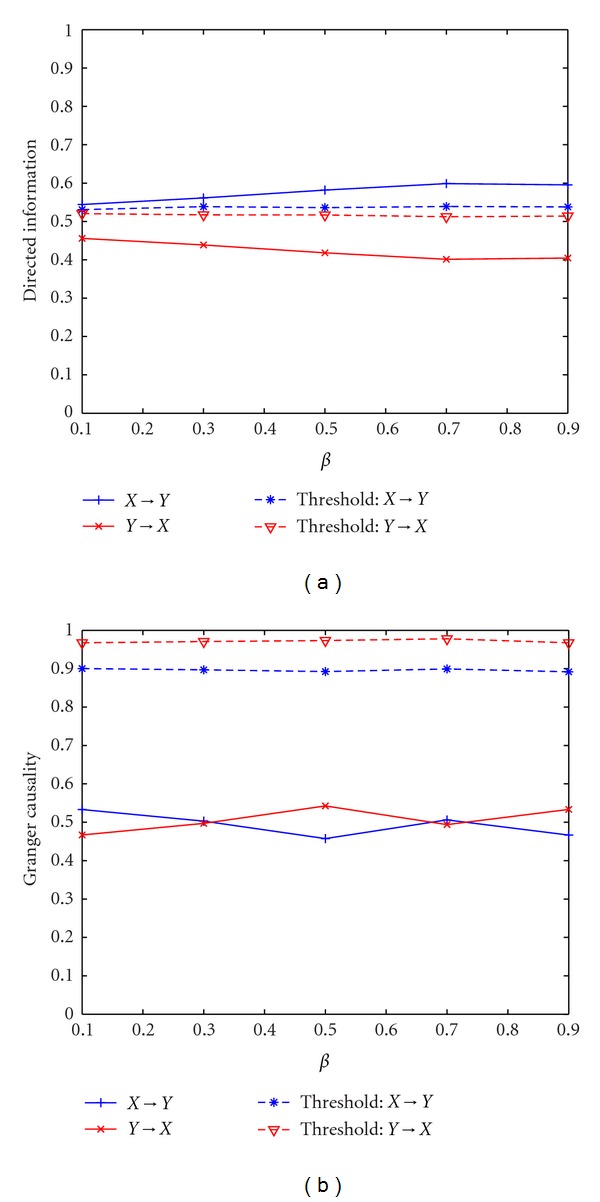
Application of directed information and Granger causality to two asymmetric coupled Lorenz oscillators. (a) Directed information with different *β*. (b) Granger causality with different *β*.

In this example, the DI values and GC values between **X**_1_ and **X**_2_ of two asymmetric coupled Lorenz systems are computed with coupling strength *β* being set from 0.1 to 1. The estimated order of the model is 3. Though this is larger than the actual model order, our method will not lose any information except for the increased computational complexity. The results are shown in Figure 5. The results show that DI values from **X**_1_ to **X**_2_ increase with the coupling strength *β* and are significant for all values of *β*. In addition, there is no significant causal information flow from **X**_2_ to **X**_1_. Therefore, DI can effectively detect the causality in a nonlinear dynamic system. On the contrary, GC cannot detect any significant information flow for all *β* values. It is due to the fact that the model selected for implementing GC is not consistent with the dynamic characteristics of the system.

### 3.2. EEG Data

**Figure 6:**
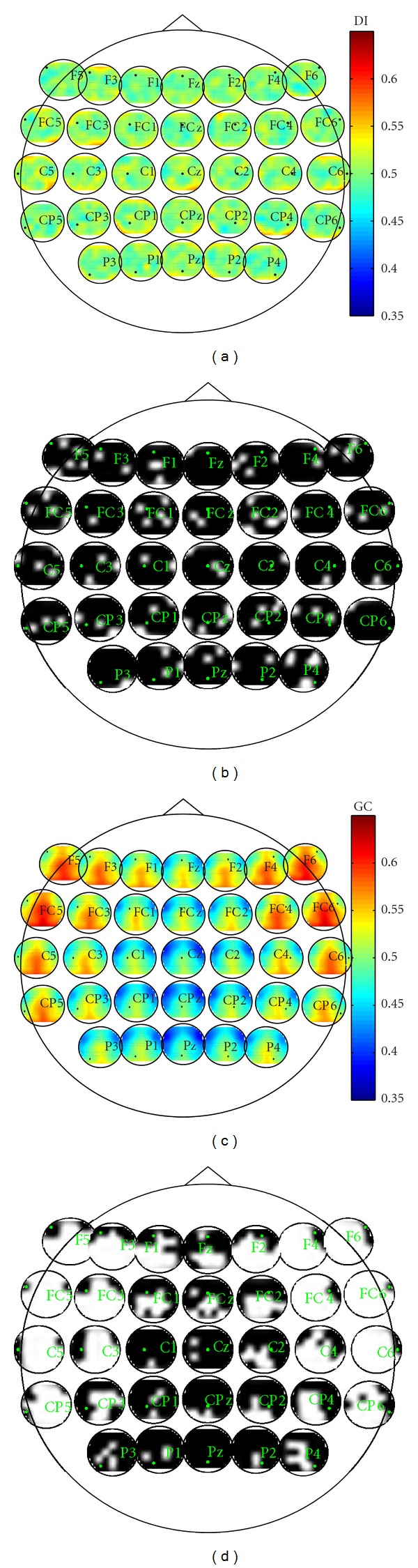
Application of directed information and Granger causality to EEG data. (a) Pairwise directed information. (b) Electrode pairs with significant DI values. (c) Pairwise Granger causality. (d) Electrode pairs with significant GC values. For (b) and (d), green dots indicate the location of the particular node, and white regions correspond to significant information flow from that particular electrode to other electrodes.

Previous work indicates that there is increased information flow associated with ERN for the theta frequency band (4–8 Hz) and ERN time window 25–75 ms for error responses compared to correct responses in particular between mPFC and lPFC regions [55]. In addition, Cavanagh et al. have shown that there is increased synchronization for error trials between electrode pairs, such as FCz-F5 and FCz-F6, compared to the synchrony between FCz-CP3 and FCz-CP4 [56]. The DI and GC values for each pair of electrodes averaged over 10 subjects are computed over a time window of 53 time points (100 ms). The estimated order of the model for each electrode pairs is 3. In order to control the error rates for multiple hypothesis testing for all pairs of electrodes, the method proposed by Genovese et al. is used in this paper [57]. To implement this procedure, for two electrodes with time series **X** and **Y**, we first shuffle the order of the trials of **X** 100 times to generate new observations **X**_m_*, *m* = 1,…, 100. The *P* value of DI(**X** → **Y**) is obtained by comparing it with DI values from randomized pairs of data DI(**X**_m_* → **Y**), *m* = 1,…, 100. We then obtain the threshold *P*_*r*_ for all *P* values (33 × 33 × 10) by controlling the FDR bound *q* as 0.05. For DI(**X** → **Y**), if the *P* value is less than *P*_*r*_, then the directed information flow from **X** to **Y** is significant; otherwise, it is not significant. Electrode pairs between which the information flow is significant in at least one of the ten subjects are shown in Figure 6(b). We also test the significance of Granger causality in the same way. When the FDR is controlled at 0.05, the information flow between electrode pairs is significant if the *P*-value of DI or GC is less than 0.01. Electrode pairs that have significant causality relationship using both measures are shown in Figure 6. In Figures 6(a) and 6(c), each small circle shows the directed information and Granger causality from a particular electrode to other electrodes. In Figures 6(b) and 6(d), each small circle shows electrode pairs that have significant causality relationship. The results indicate that DI detects strong information flow from the frontal region (e.g., F5 and F6) to the frontal-central region (e.g., FC2 and FCz) corresponding to the lateral prefrontal cortex (lPFC) and medial prefrontal cortex (mPFC). In addition, the central-parietal region (e.g., CPz, CP1, and CP2) around the midline, corresponding to the motor cortex, has strong influence on the central and frontal regions (e.g., FCz and F6) since this is a speeded response task involving the motor cortex. The details of the significant electrode interactions are shown in Table 1. These results are aligned with the previous work in [56], which shows that error processing is controlled by the communication between the lateral prefrontal cortex and medial prefrontal cortex. When GC is applied to the same data, the information flow pattern around the midline is similar to the DI. However, the information flow from the lateral prefrontal cortex to the rest of the brain is significant. On one hand, the similar patterns of connectivity using both measures verify the validity of proposed DI computation algorithm. On the other hand, GC shows significance over a wide region of the brain especially in the lateral areas compared to DI, which may be due to GC's low specificity to volume conduction in the form of a common source. Previous work and our simulation in Example 4 have indicated that Granger-causality-based measures may infer erroneous effective connectivity in the case of the common source as seen in EEG data [19, 46]. However, without ground truth, we cannot confirm that some links reported as significant by GC are spurious and due to volume conduction in a conclusive manner, but the results from DI agree more with the suggestions in [56], that most of the increase in connectivity during cognitive control, that is, ERN, should be between medial prefrontal cortex and lateral prefrontal cortex, compared to the results of GC. Therefore, DI is more sensitive and discriminative about the information flow patterns compared to GC for real neurophysiological data.

## 4. Conclusions

In this paper, we illustrated the advantages of a new directed information measure over Granger-causality-based measures for quantifying the effective connectivity in the brain. In order to illustrate the advantages of this measure, first, we applied directed information measure to identify the causality relationships for both linear and nonlinear AR models, linear mixing models, single source models, and Lorenz systems and compare its performance with Granger causality. Directed information is shown to be more effective in detecting the causality of different systems compared to Granger causality. We then applied the directed information measure on EEG data from a study containing the error-related negativity to infer the information flow patterns between different regions. The results showed that the directed information measure can capture the effective connectivity in the brain between the mPFC and lPFC areas as predicted by previous work.

Directed information, as a model-free measure, is able to detect both linear and nonlinear causality relationships between two signals. However, other model-free entropy-based measures would also detect effective connectivity such as transfer entropy and directed transinformation. Directed transinformation introduced by Saito measures the information flow from the current sample of one signal to the future samples of another signal given the past samples of both signals but does not discriminate between totally dependent and independent processes. Transfer entropy and directed information are very closely related to each other. Transfer entropy quantifies the information gained at each time step by measuring the deviation of the observed data from the generalized Markov condition. Therefore, the definition of transfer entropy implicitly assumes a stationary Markov process [31]. Compared to transfer entropy, directed information quantifies the sum of information obtained over the whole time series [58] and does not make any assumptions about the underlying signal model. Thus, theoretically, the original definition of directed information can apply to any signal models. In real applications, in order to simplify the computation of directed information, we usually make certain assumptions about the underlying signal model such as the modified time-lagged DI proposed in this paper, which basically assumes a stationary Markov process similar to transfer entropy. In addition, Amblard and Michel proved that, for a stationary process, directed information rate can be decomposed into two parts, one of which is equivalent to the transfer entropy when *l* = *m* = *n* in (1) and the other to the instantaneous information exchange rate [31]. In another words, for a physical system without instantaneous interactions between its subsystems, the rate of these two measures, directed information and transfer entropy, is equivalent asymptotically as the length of the signal goes to infinity.

There are still remaining issues with the implementation of directed information. First, the performance of directed information relies on accurate estimation from limited sample sizes that introduces bias to the estimated values. This problem can be addressed by either using parametric density models or improving existing mutual information and entropy estimators. Recently, Zhao et al. proposed an universal algorithm to estimate directed information for stationary ergodic processes by using sequential probability assignment, which may be used to improve the effective connectivity results discussed in this paper [59]. Second, the performance of directed information relies on the selection of the model order. If the order of the model is too small, it will lose the information from **X** to **Y**. If it is too large, the computational complexity is very high. In addition to classical embedding dimension determination methods such as the Cao criterion used in this paper, Faes et al. proposed a sequential procedure to determine the embedding dimension of multivariate series [60]. This method is based on an information-theoretic technique and shows promising performances for various signal models, which may be extended to DI computation in the future. Third, directed information does not discriminate between direct and indirect interactions among multivariate time series. However, this is not a shortcoming of DI since DI does not assume any particular signal interaction model: bivariate or multivariate. Similar to other information theoretic measures, such as mutual information, whether the particular measure can identify interactions between multiple processes depends on how the measure is applied. For example, in the case of mutual information, though the original definition is for two random processes **X** and **Y**, it is possible to extend it to multiple processes [61]. Similarly, we can apply DI over multiple processes using conditional directed information such as the definition given by Kramer. We address this issue in a previous paper [34] by using conditional directed information and develop an algorithm to infer the actual network. Similarly, GC originally is defined for two time series that a stochastic process **X** causing another process **Y** if the prediction of **Y** at the current time point, *Y*_*n*_, is improved when taking into account the past samples of **X**. However, in application it has been extended to multiple processes through the use of multivariate AR models. Future work will focus on the comparison of these two measures in a multivariate setting.

## Figures and Tables

**Table 1:** Electrode pairs in the region of interest with significant DI values.

From	To	From	To	From	To
F5	F1 FC2 CPz CP4 P3	C5	F6 FC5 Cz CP4	P3	P4
F3	FC3 CP4	C3	FC2 C6 P1	P1	F2 C6 CP2
F1	C1 Cz Pz	C1	FC1 C6	Pz	F5 F4 FCz
FZ	F5	CZ	F5 C2 CP4	P2	FC4 C5
F2	FC3 FC6 C5 CP1	C2	FC6	P4	F3 F4 FC3 FC2 FC4 Pz P2
F4	F6 C4	C4	P2		
F6	F2 FC3 FCz Cz	C6	Pz		
FC5	Fz C3 C2 CP6	CP5	Cz C4 CP3		
FC3	CP1	CP3	C5 CPz P4		
FC1	F4 FC3 C2 CP1 CP4	CP1	F6 FCz P3		
FCZ	C3 CP1	CPz	FC6 C6 CP5 CP4 P1		
FC2	F3 C1 C6 CP2 CP4 P3	CP2	F6 FCz FC4 CP1		
FC4	C5	CP4	FC5 FCz C4		
FC6	C5 C4 CP1	CP6	F5		
